# An Intriguing Case of Gynecomastia in an Elderly Male as the Initial Presentation of Graves’ Disease: A Case Report

**DOI:** 10.7759/cureus.39286

**Published:** 2023-05-21

**Authors:** S.L. Sravya, Jayshree Swain, Kasukurti Lavanya, Brij R Teli, Pooja A Jadhao

**Affiliations:** 1 Endocrinology, Diabetes and Metabolism, Institute of Medical Sciences and SUM Hospital, Siksha 'O' Anusandhan Deemed to be University, Bhubaneswar, IND

**Keywords:** shbg(sexhormone binding globulin), testosterone, elderly male, gynecomastia, graves’ disease

## Abstract

Gynecomastia is the proliferation of fibroglandular tissue in the male breast due to an altered hormonal milieu between the inhibitory effect of androgens and the stimulatory effect of estrogens on the breast tissue causing feminization of the male breast. Physiological causes are more common along with a few pathological conditions leading to gynecomastia in the male population. Of these varied etiologies, thyrotoxicosis is one of the notable causes, though it is very rare in the elderly population. Gynecomastia as the initial presentation of Graves’ disease that too in an elderly age group is very rare with very few cases reported in the literature. Here, we present a case of a 62-year-old male presenting with gynecomastia, who after a detailed evaluation, was diagnosed to have Graves’ disease.

## Introduction

Gynecomastia in the male population has a varied prevalence ranging from 32 to 65% depending on age and lifestyle factors [1,2]. Multiple etiologies are attributed to gynecomastia ranging from physiological to pathological causes and benign to malignant causes. Of these, thyrotoxicosis has been one of the attributable causes. Thyrotoxicosis as an etiology of gynecomastia is rare contributing to less than 1.5% of the causes [3]. The prevalence of gynecomastia in thyrotoxicosis ranges from 10 to 40% of the cases [4]. However, gynecomastia being the initial presenting feature of thyrotoxicosis in an elderly male is very rare, with only a few case reports in the literature.

## Case presentation

**Figure 1:**
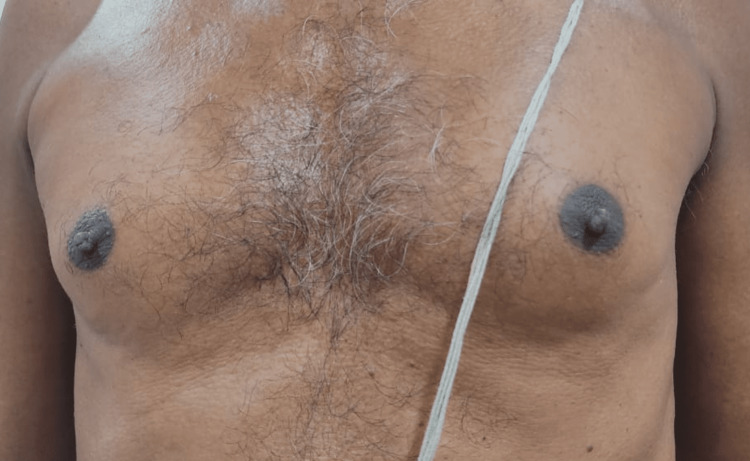
Anterior view of both breasts.

**Figure 2:**
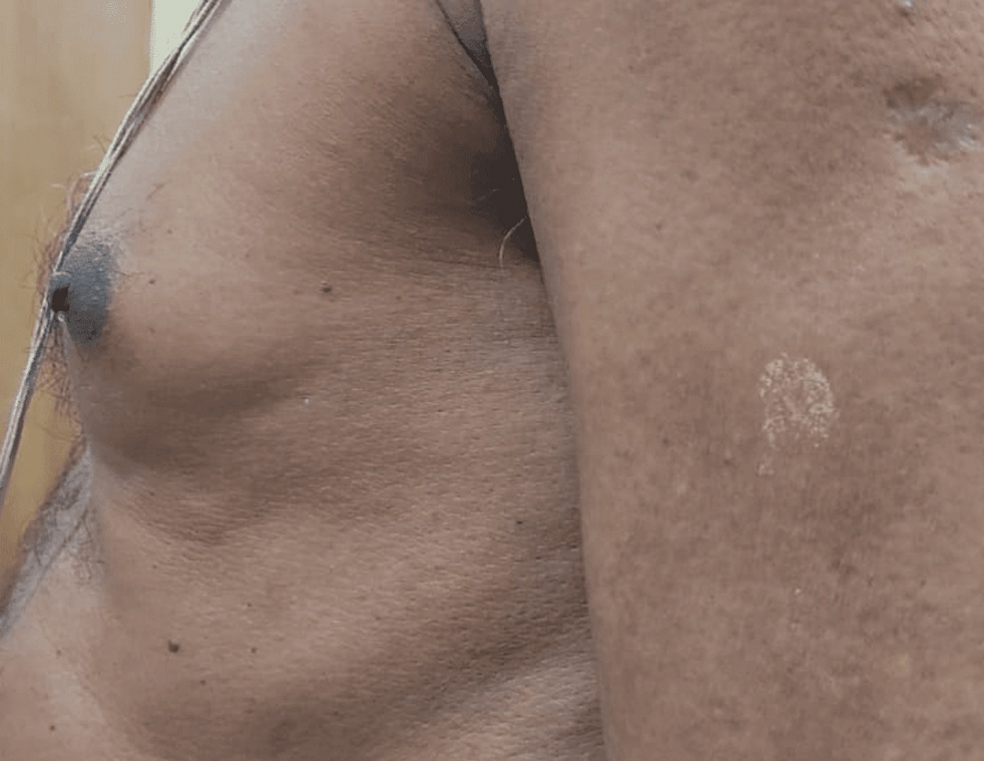
Lateral view of the left breast

**Figure 3:**
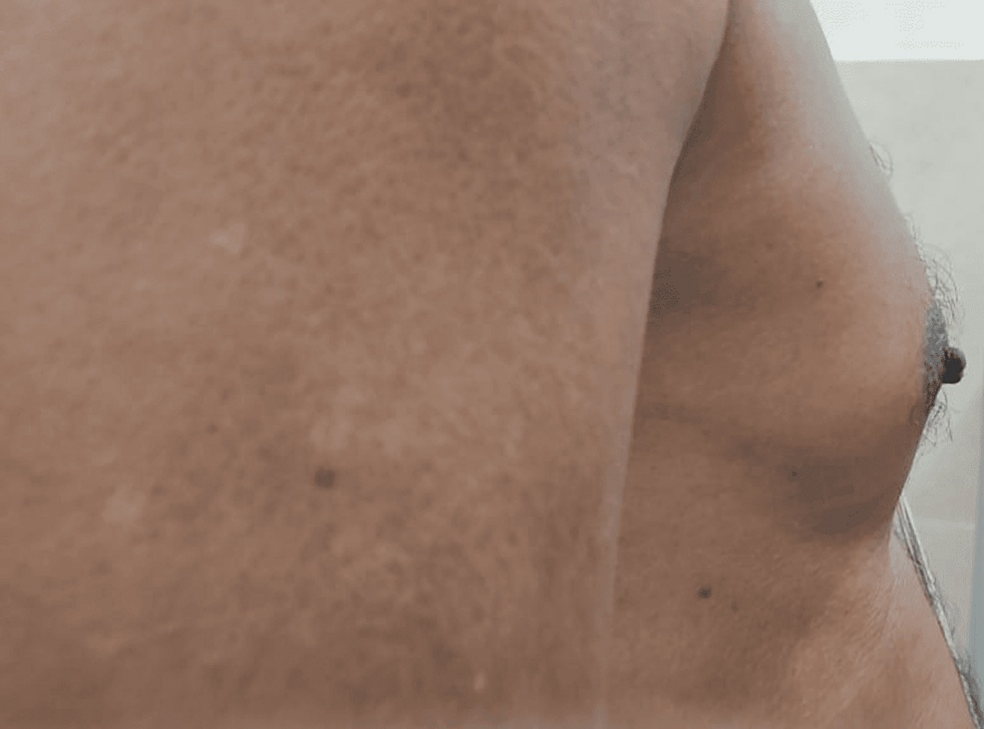
Lateral view of the right breast

A 62-year-old male with no comorbidities presented to our endocrinology outpatient department with bilateral painful gynecomastia with the size of the left more than the right breast for three months duration. It was gradually increasing in size, with no signs of local inflammation. On detailed inquiry, he had a history of palpitations, eye discomfort, tremors, and weight loss of 3kgs in the past six months. There was no history of similar episodes in the past, no history of illicit drugs or alcohol abuse, and no history of medication intake (ACE inhibitors, ketoconazole, spironolactone, tricyclic anti-depressants, testosterone replacement, anabolic steroids, etc). History was not suggestive of underlying systemic illness (with no stigmata for chronic kidney or liver disease) or malignancy. There was no history suggestive of hypoandrogenism (decreased frequency of shaving, decreased libido). His family history was not significant. On physical examination, he was normally built with a BMI of 21kg/m^2^. Vital signs revealed the following: temperature, 98.6 °F; pulse, 118 bpm (high-volume pulse); blood pressure, 138/74 mmHg; and SpO_^2^_, 97% in room air. Bilateral breasts showed tender fibroglandular tissue with a disc diameter measuring three and a half centimeters in the right and about five centimeters in the left breast with Cordova & Moschelle grade II Gynecomastia (Figures 1-3). No palpable lymph nodes were present. Thyroid examination was suggestive of Grade I, diffuse, soft, and nontender goiter. Fine tremors were noticed in the outstretched moist hands. There was no evidence of exophthalmos or active orbitopathy, and the clinical activity score was noted to be one out of seven, with no signs of dermopathy or acropachy. His secondary sexual characteristics were normal with a stretched penile length of 12 centimeters, bilateral testicular volume of 20 cubic centimeters with normal consistency, and no palpable mass. Systemic examination was not suggestive of organomegaly or palpable mass, with no other features suggestive of malignancy. Nervous system examination was otherwise normal except for fine tremors and mild proximal myopathy of lower extremities.

**Figure 4:**
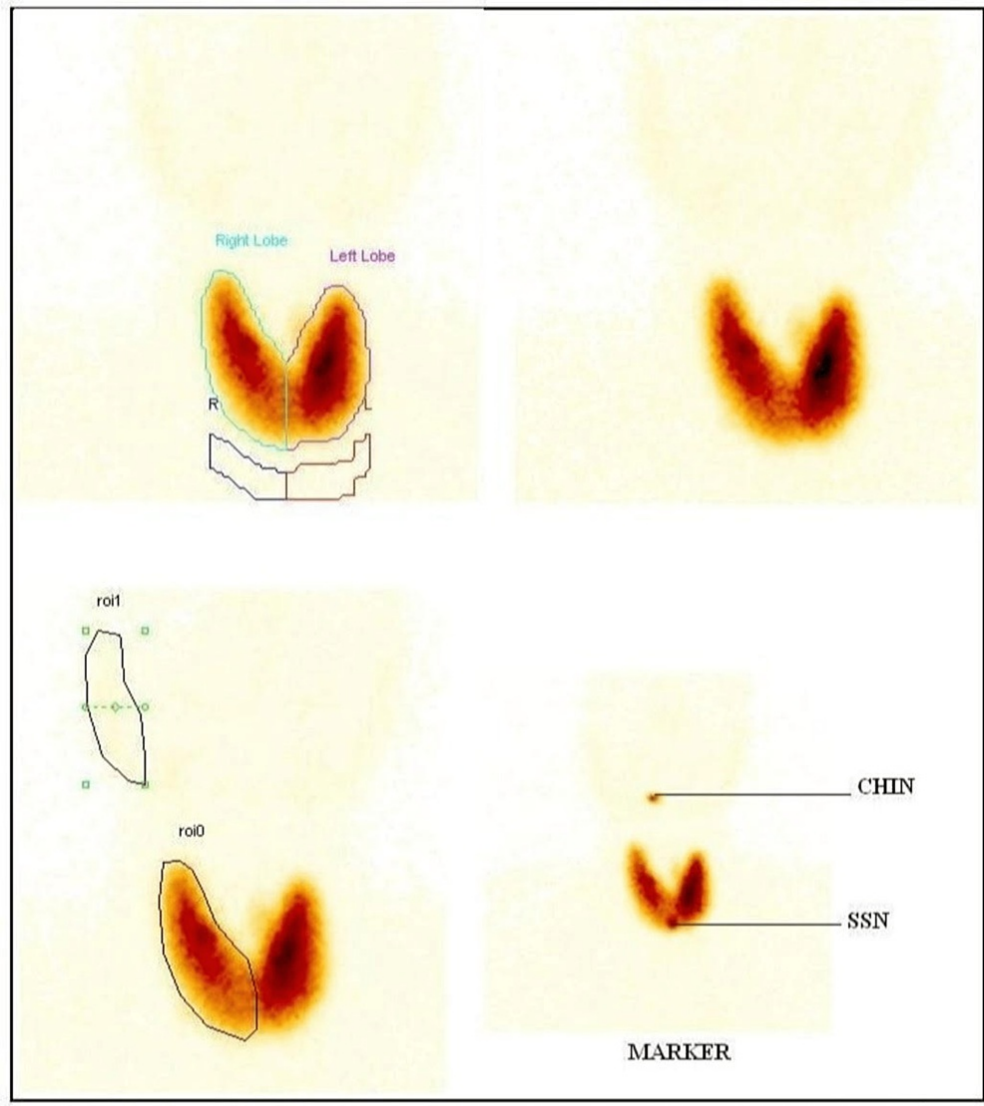
Technetium 99m thyroid uptake scan-uptake : 30.8%

His routine investigations like plasma glucose, liver function, renal function test, erythrocyte sedimentation rate, and complete blood picture were normal. Total T3 is 3.47ng/ml, total T4 is 16.2µg/dl, TSH is <0.005µIU/ml, and anti TPO is 761IU/ml. Total testosterone is 428ng/dl, Sr. Estradiol is 75 pg/ml. β-hCG is 132nmol/L, and FSH, LH, and prolactin levels were within normal limits. Other biochemical investigations of interest are shown in Table 1. Ultrasonography of the breast showed fibroglandular tissue in bilateral breasts. Fine needle aspiration cytology confirmed the presence of breast tissue with no features suggestive of malignancy. Ultrasonography of the abdomen and scrotum was normal. Chest x-ray was normal. Ultrasonography of the thyroid was suggestive of a diffusely enlarged thyroid with increased vascularity (peak systolic velocity in the inferior thyroid artery - 60cm/s). Technetium 99 scan showed diffuse uptake (Figure 4). With this evidence, he was diagnosed to have Graves’ disease with gynecomastia.

**Table 1:** Biochemical investigations of the patient T3: Triiodothyronine, T4: thyroxine, TSH: thyroid stimulating hormone, TPO: thyroxine peroxidase, SHBG: sex hormone binding globulin, β-hCG: beta-human chorionic gonadotropin, FSH: follicular stimulating hormone, LH: luteinizing hormone

Blood investigation	Report	Reference range
T3	3.47ng/ml	0.8- 2.0 ng/ml
T4	16.2µgm/dl	5.1-14.06µg/dl
TSH	<0.005µIU/ml	0.5-8.9 µIU/ml
Anti TPO antibody	761 IU/ml	<50 IU/ml
Total testosterone	428ng/dl	
SHBG	132 nmol/L	7-50 nmol/L
β-hCG	<0.2mIU/ml	<5mIU/ml
Estradiol	75pg/ml	<50pgm/ml
FSH	7.8mIU/ml	0.7-11.1 mIU/ml
LH	6.4mIU/ml	0.8-7.6 mIU/ml
Prolactin	14 ngm/dl	4.7-15.2 ngm/dl

 

**Figure 5:**
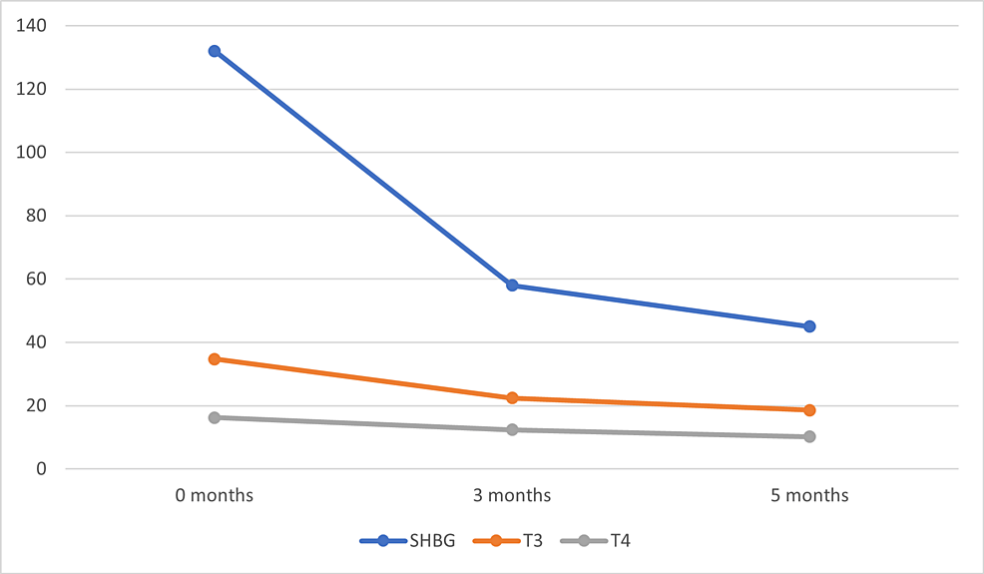
Serial thyroxine (T4) and triiodothyronine (T3) levels in comparison to sex hormone-binding globulin (SHBG) in follow up T4: Thyroxine; T3: triiodothyronine; SHBG: sex hormone binding globulin

He was started on propranolol and carbimazole 30mg per day in divided doses. On subsequent follow-up at two months, general well-being was improved with weight gain. The thyrotoxic features subsided with the normalization of T3 and T4, and with a very mild reduction in gynecomastia (Table 2, Figure 5). The carbimazole dose was reduced to 20mg/day and was well tolerated with no documented adverse effects. At the fifth-month follow-up, gynecomastia regressed in size by more than half with normalization of Sex hormone binding globulin (SHBG) and thyroid profile. After one year, there was no further change in the size of the breast tissue. The carbimazole dose was further tapered and was advised for regular follow-up.

**Table 2:** Serial biochemical investigations of the patient T3: Triiodothyronine; T4 - thyroxine; TSH - thyroid stimulating hormone; SHBG - sex hormone binding globulin

Parameter	At diagnosis	2 months	5 months
T3	3.47 ng/ml	2.24 ng/ml	1.86 ng/ml
T4	16.2 µgm/dl	12.4 µgm/dl	10.2 µgm/dl
TSH	<0.005 µIU/ml	<0.01 µIU/ml	0.1 µIU/ml
Total Testosterone	428 ng/dl	448 ngm/dl	567ng/dl
SHBG	132 nmol/L	58 nmol/L	45 nmol/L

## Discussion

Gynecomastia seen in elderly male patients is mainly physiological due to andropause. Another important cause is malignancy. However, gynecomastia being the initial presentation of Graves’ disease is very rare. Only very few case reports of gynecomastia as a presenting feature of Graves’ disease are found in the literature, most of them in the middle age group patients. Male gynecomastia is caused due to the altered hormonal milieu of free testosterone and estrogen ratio rather than the absolute levels. As in our case, total testosterone is within normal limits, but the patient has a low calculated free testosterone to estrogen ratio.

Few mechanisms were proposed for the development of gynecomastia in thyrotoxicosis. Firstly, SHBG is the major binding protein of both testosterone and estrogen but has a high affinity to testosterone [5]. High levels of thyroid hormones stimulate SHBG production from the liver. The probable mechanism by which thyroid hormones increase the SHBG is through HNF4α (hepatocyte nuclear factor 4α) pathway [6]. This results in decreased free testosterone levels and an altered estrogen-to-testosterone ratio. The second mechanism is the thyroid hormones increase the activity of aromatase that converts androgens to estrogens [7]. This increases the estrogen levels altering the estrogen-to-testosterone ratio. The third is increased levels of metabolites of dehydroepiandrosterone and DHEAS-androstenediol (5-androstane-3𝛽, 17∼𝛽-diol, androstenediol 3-sulfate). Though the estrogenic activity of these compounds is low, their higher serum concentrations contribute to their estrogenic action [8].

With the initiation of treatment in due course, these mechanisms are slowly reversed and normal hormonal milieu is established and breast tissue regresses with time. As seen in our patient, after the initiation of antithyroid therapy, the breast tissue has regressed by more than 50% after six months with no further regression noted after a year, whereas few case reports documented complete regression. Our study was different from previous case reports as most of the previous studies have been reported in the middle age group. Only Jayapaul et al. reported a similar case in an 84-year-old male [9]. Few cases were reported with additional features of hypogonadism like loss of libido and impotence, which was not documented in our case [10]. Galactorrhea is a very rare presentation in a few case reports, not present in our case [11]. Proximal muscle weakness is a well-documented association with Graves’ disease, which was noted in our case, and it gradually improved with treatment [12]. Both unilateral and bilateral gynecomastia were reported in the literature [13-15]. Presentations in previous case reports were compared in Table 3.

**Table 3:** Comparison of presentations of previous case reports FT3 - Free Triiodothyronine; FT4 - free thyroxine; SHBG - sex hormone binding globulin

Author	Age (yrs)	Duration (months)	Presenting features	Outcome
Tauheed et al. 2002 [12]	52	2	Unilateral painful gynecomastia and proximal lower-limb weakness	Gynecomastia resolved in two months
Sanyal et al. 2012 [13]	35	1	Bilateral painful gynecomastia	
Wang et al. 2013 [14]	39		Unilateral gynecomastia and hypokalemic periodic paralysis	Euthyroid state in four months and gynecomastia resolved in 14 months
Sakulterdkiat et al. 2015 [10]	56	6	Unilateral painful gynecomastia, decreased libido, and impotence	3 months- Euthyroid, SHBG, and testosterone normalized gynecomastia reduced in size
Khoohaphatthanakul et al. 2016 [11]	33	2	Bilateral galactorrhea	3 months- Euthyroid, galactorrhea subsided five months- gynecomastia resolved
Mohammadnia et al. 2021 [15]	49	3	Unilateral gynecomastia and decreased libido	1 month -FT3/FT4 normalized two months- SHBG & Testosterone normalized three months – gynecomastia & libido improved

Direct free testosterone measurement was not performed due to non-feasibility, but free testosterone was calculated as a surrogate marker. Other tumor markers and high-end investigations for malignancy screening were not done as clinically not warranted.

## Conclusions

Graves’ disease with the initial presenting feature as gynecomastia is very rare in the elderly age group population. Hence, it should be considered as one of the differential diagnoses while evaluating a case of gynecomastia in the elderly population, before attributing it to be either age-related physiological gynecomastia or evaluating extensively for malignancy. Treatment of Graves’ disease with antithyroid drugs usually improves or resolves the gynecomastia.
